# Different judgment frameworks for moral compliance and moral violation

**DOI:** 10.1038/s41598-024-66862-9

**Published:** 2024-07-16

**Authors:** Risako Shirai, Katsumi Watanabe

**Affiliations:** 1 Faculty of Science and Engineering, Waseda University, 3-4-1 Okubo, Shinjuku-ku, Tokyo, 169-8555 Japan; 2 Japan Society for the Promotion of Science, Tokyo, Japan

**Keywords:** Psychology, Human behaviour

## Abstract

In recent decades, the field of moral psychology has focused on moral judgments based on some moral foundations/categories (e.g., harm/care, fairness/reciprocity, ingroup/loyalty, authority/respect, and purity/sanctity). When discussing the moral categories, however, whether a person judges moral compliance or moral violation has been rarely considered. We examined the extent to which moral judgments are influenced by each other across moral categories and explored whether the framework of judgments for moral violation and compliance would be different. For this purpose, we developed the episodes set for moral and affective behaviors. For each episode, participants evaluated valence, arousal, morality, and the degree of relevance to each of the Haidt's 5 moral foundations. The cluster analysis showed that the moral compliance episodes were divided into three clusters, whereas the moral violation episodes were divided into two clusters. Also, the additional experiment indicated that the clusters might not be stable in time. These findings suggest that people have different framework of judgments for moral compliance and moral violation.

## Introduction

We routinely see conflicts of opinion between individuals or groups on social networking sites, television, and newspapers (e.g., wearing mask on a train, unfairness dress code on gender, and movements about induced abortion). Such individual differences in moral judgments sometimes escalate to discrimination or more violent conflict.

To understand the mechanisms of moral judgments, researchers in moral psychology have studied the principles of human morality. Recently, the model positing an innate moral principle has gained attention^1,2^. For example, the Universal Moral Grammar model is characterized by that humans have innate moral faculties like the Chomsky's idea^3^ about linguistics^1,2^. Further, the Moral Foundations Theory (MFT;^4,5^) explains where the specific moral intuitions come from and includes the sociohistorical elements and social interactions that cannot be fully explained within a general cognitive framework^2^. Specifically, Haidt^6^ proposed the MFT^4,5^ that the moral judgments are primary determined by the autonomic intuition process rather than the conscious reasoning process. MFT argues that this intuition is supported by the five foundations or bases (follow harm/care, fairness/reciprocity, ingroup/loyalty, authority/respect, purity/sanctity) and the differential sensitivities in each moral foundation lead to the individual differences in moral judgments. Haidt and Joseph^7^ further argued that each foundation has specific psychological process and own evolutionally history. *Care/Harm* is related to the moral goods of care and kindness for suffering in others. *Fairness/reciprocity* concerns the cheating or the cooperation in reciprocal interactions. *Ingroup/loyalty* is related to the moral goods of loyalty and patriotism for the in-group. *Authority/respect* is related to the respect and owe for the authority. *Purity/sanctity* is related to the chastity and purity for the bodily and spiritual activities. However, how many foundations exist for morality is still not clear. For example, liberty/oppression is now also included as a basis for the sixth foundation^8^. Moreover, equality and proportionality (e.g.,^9^), honor^10^, and ownership^11^ have been proposed as candidate domains or in the place of the “*Fairness/reciprocity*” domain (e.g.,^9^). Further, Curry, Mullins, and Whitehouse^12^ reported the seven types of cooperative behaviors for universal moral rules.

For the number of the moral domains, the researchers suggest that morality can be divided into three (i.e., autonomy, community, and divine ethics;^13^), five, or six categories^7^, or even primary explained by one component (e.g., *Harm* component^14,15^). Haidt and Joseph^16^ noted that it is important to advance the understanding of moral functioning by counting and observing the distinctive moral content. However, in response to the methodology of Haidt and Joseph^16^, Carchidi^2^ featured the notion of Chomsky^17^ that it is difficult to effectively understand diversity by skipping understanding the mechanisms underlying moral judgments.

For exploring the structure of moral foundations, several useful episode sets have been developed (e.g., moral foundations vignettes: MFVs^18^; Moral Foundations Dictionary: MFD^19^; Japanese versions of MFD: J-MFD^20^; Socio-Moral Image Database: SMID^21^; Moral and Affective Film Set: MAAFS^22^). However, many previous studies used episodes depicting an act(s) of moral violation, as opposed to moral compliance (or adherence to the moral rules or virtues). Moreover, even when moral compliance behaviors were included, separate analyses for moral compliance and moral violation were not performed. For example, hurting and caring for others are both considered to be associated with a *Care/Harm* foundation (e.g.,^23^). Recently, studies have focused on the compliance side of morality. Curry et al.^12^ suggested that morality as cooperation can predict a broader moral phenomenon than the other accounts of morality and reported that seven cooperative behaviors were evaluated as morally good for 60 diverse societies. Further, emotional studies have argued that positive and negative emotions are relatively independent (e.g.,^24–27^). For instance, previous studies suggested that the negative and positive affects facilitated problem-solving (e.g.,^26^) in different ways and differently affected job performances^27^. Moreover, Cunningham, Steinberg, and Grev^28^ reported that positive moods and negative events (guilt-related) increased helping behaviors under different conditions. Given the differences between positive and negative functions, the studies of moral judgments will require the accumulation of data on both compliance and violation.

Here, we focused on how moral judgments are exclusive across moral categories. Examining the extent to which moral categories could be subjectively distinguished, we considered that it would be possible to explore whether the framework of moral judgment is multidimensional, at least in terms of explicit judgments. Moreover, we focused not only on the aspect of moral violation but also on the aspect of moral compliance to explore whether the framework of judgments for moral violation and compliance would be different.

For this purpose, we developed a collection of episodes of moral violation and moral compliance associated with various moral contents. To cover the diverse and complex moral judgments, we referenced the Haidt’s taxonomy of morality. First, a total of 390 episodes of moral violation, moral compliance, and affective but moral-neutral related behaviors were created. Then, participants rated the valence, arousal, and the degree of morality of the main character in the episode. They also rated how relevant the episode was to contents of each of the five moral foundations using a 0–100% scale. Based on the degree of relevance of each episode to the five moral foundations, we performed cluster analyses separately for the moral violation episodes and the moral compliance episodes.

## Experiment 1

### Methods

The codes of the analysis and episodes set are available and can be accessed at [https://osf.io/jtxyp/?view_only=63607826eed24593a4a7c7f0337b0b62].

#### Sample

Participants were recruited through the Yahoo crowdsourcing system (https://crowdsourcing.yahoo.co.jp/; January 25th to 26th, 2021). Considering the validity and reliability of the ratings per episode and the crowdsourcing system’s limitation, the participants were recruited so that the number of participants rating each episode would be at least 100. Finally, 1555 participants (599 women, 956 men, mean age = 45.57 years, age range = 18–78 years) participated in the study. All participants gave their informed consent over the Internet before participating in all experiments in our study. Ethical approval for our study was obtained by the Ethics Review Committee on Research with Human Subjects in Waseda University, and the study was conducted in accordance with relevant guidelines and standard including the Ethical Guidelines for Medical and Biological Research Involving Human Subjects and the Declaration of Helsinki. The present study was not preregistered.

#### Instrument

One of the authors created 390 episodes with reference to the episodes of Anderson, Siegel, Bliss-Moreau, and Barrett^29^ and Konishi, Oe, Shimizu, Tanaka, and Ohtsubo^30^, which included the part of episodes from Shirai and Ogawa^31^. There were 13 types of episodes (moral compliance: care, fairness, authority, ingroup, purity; moral violation: harm, unfairness, despise, betrayal, impurity; affective but moral-neutral: positive, negative, neutral) and 30 sentences for each type. Finally, due to technical problems, the following episode categories resulted in changes in the number of episodes (29 *Fairness* episodes, 31 *Unfairness* episodes, and 29 *Positive* episodes were used). All the episodes included the contents describing the actions of a person. The episodes of each type were created by follows. Episodes of *Care* were created to include keywords related to protecting and helping others. Episodes of *Fairness* were prepared to relate to cooperation and equality. Episodes of *Authority* were designed to relate to the respect and defense for the superior and its symbols. Episodes of *Ingroup* were created to include the acts of respect for the in-group members. Episodes of *Purity* were designed to relate to the acts such as valuing the chastity and purity and avoiding contamination. Episodes of *Harm* were created to include the acts associated with the physically or mentally harming others. Episodes of *Unfairness* were prepared to include the keywords related to cheating others and unequal behavior. Episodes of *Despise* were created to include the acts related to disobeying a superior and defiling its symbols. Episodes of *Betrayal* were created to include the acts related to betraying in-group members. Episodes of *Impurity* were designed to include the acts related to impurity such as staining the sacred objects. The affective but moral-neutral episodes were created without considering the five moral foundations: Episodes of *Positive* category were designed to include the good things that happen independent of social interactions with others. Episodes of *Negative* category were designed to include bad events that occur independently of interactions with others. Episodes of *Neutral* category were created to include events in daily lives that do not seem evoke emotions strongly. Each episode was created by referring to events that occurred in daily lives or were reported in news reports.

#### Procedure

All participants completed the experiment online through the Yahoo crowdsourcing system. The experiment was created by Qualtrics (qualtrics.com). In the experiment, 26 episodes were shown on the display in sequence with the questionnaire. Participants were asked to read the episode and rate the valence (1 = *extremely unpleasant*, 9 = *extremely pleasant*) and arousal (1 = *extremely low*, 9 = *extremely high*) for the episode, the morality of the main character in the episode (1 = *extremely moral violation acts*, 9 = *extremely moral compliance acts*) and how much the content of the episode related to each moral category in percentages (Supplementary Material). The episodes rated by the participants were designed to include two episodes for each type of episode (i.e., 10 moral compliance episodes: 2 care, 2 fairness, 2 authority, 2 ingroup, 2 purity; 10 moral violation episodes: 2 harm, 2 unfairness, 2 despise, 2 betrayal, 2 impurity; 6 affective but moral-neutral episodes: 2 positive, 2 negative, 2 neutral), for a total of 26 episodes evaluated. The episodes were divided into 15 sets of 26 episodes each, and participants rated the episodes from one of the sets. The 26 episodes in each set were presented in random order. There was no time limit for each question.

### Results

#### Moral and affective episodes set (MAES)

We developed the set of moral compliance, moral violation, and affective but moral-neutral related episodes to understand the structures of moral categories. Table 1 shows the basic demographics in Experiment 1. Tables 2 and 3 show that summary descriptive statistics for the episodes. The episodes and the ratings for each episode are available on the OSF (https://osf.io/jtxyp/?view_only=63607826eed24593a4a7c7f0337b0b62). The descriptive statistics showed that the moral compliance episodes were evaluated as more moral than the moral violation episodes. The episodes of positive category were rated as more positive and the episodes of negative category were rated more negative, and the episodes of neutral category were rated as the median value among the scale. These confirmed the face validity of the episodes in each category (i.e., moral compliance, moral violation, affective but moral-neutral). On the ratings of each moral category, the episodes of *Care* category were rated as more positive, and the episodes of *Harm* category were evaluated as more negative than the episodes of other categories. Also, the episodes of *Care*/*Harm* categories were rated as the highest arousal among the episodes of other categories. Moreover, the episodes of *Care* category were rated as more moral and the episodes of *Harm* category were rated as more immoral than the episodes of other categories. We conducted the Kruskal–Wallis test for mean valence with each factor (5 moral categories: *Harm*/*Care*, *Fairness*/*Unfairness*, *Ingroup*/*Betrayal*, *Authority*/*Despise*, *Purity*/*Impurity* or 2 moral directions: moral violation and moral compliance) by using JASP^32^. Results showed that the mean valence was significantly influenced by the moral directions*, H* (1) = 224.08, *p* < 0.001, but not affected by the moral categories, *H* (4) = 0.18, *p* = 0.996. Dunn’s post hoc comparisons showed that the mean valence for moral compliance episodes was significantly higher than those for moral violation episodes (*p* < 0.001). Also, the mean valence of the moral violation episodes was affected by the moral categories, *H* (4) = 34.41, *p* < 0.001. Pairwise comparisons showed that *Harm*-related episodes were rated more negative than the other episodes (unfairness: *p* = 0.004, despise: *p* < 0.001, betrayal:* p* < 0.001, impurity: *p* = 0.002). There were no significant differences between other episode pairs (*p*s > 0.494). Moreover, the moral categories affected the moral compliance episodes,* H* (4) = 34.19, *p* < 0.001. The *Care*-related episodes were rated more positive than the *Authority*-related, *Ingroup*-related, and *Purity*-related episodes (*p*s < 0.001). There were no significant differences between other episode pairs (*p*s > 0.074). Next, we examined the effect of the moral categories or the moral direction on the mean arousal scores. Results showed that the moral categories affected the mean arousal,* H* (4) = 91.42, *p* < 0.001, but not the moral direction,* H* (1) = 1.24, *p* = 0.266. *Care*/*Harm*-related episodes were rated as high arousal compared to the other types of episodes (*p*s < 0.001). Authority/Despise-related episodes were rated as high arousal than the *Fairness*/*Unfairness*-related and *Purity*/*Impurity*-related episodes (*p* < 0.001; *p* = 0.048). For moral violation episodes, the moral categories significantly affected the arousal scores,* H* (4) = 53.90, *p* < 0.001. Pairwise comparisons showed that *Harm*-related episodes were evaluated as high arousal compared to other moral categories (*p*s < 0.001). For moral compliance episodes, the moral categories significantly affected the arousal scores,* H* (4) = 53.90, *p* < 0.001. Pairwise comparisons showed that *Care*-related episodes were evaluated as high arousal compared to *Authority*-related, *Ingroup*-related, and *Purity*-related episodes (*p*s < 0.001). Also, *Fairness*-related episodes were rated higher arousal than *Authority*-related episodes (*p* < 0.001) and *Purity*-related episodes (*p* = 0.007). These findings indicated that the individuals were highly sensitive especially to the contents related to the *Care*/*Harm* category.

**Table 1:** Basic demographics in Experiment 1.

Experiment 1	*n*	Range	*Mean*	*Median*	*SD*	Gender	
						Women	Men
Emerging adult	73	18–25	21.67	22	2.23	48	25
Young adult	372	26–39	33.91	35	3.85	198	174
Middle adult	946	40–59	48.53	48	5.24	308	638
Late adult	164	60–78	65.53	65	4.62	45	119
Total	1555		45.57	46	11.38	599	956

**Table 2:** Mean valence, arousal and morality for each moral compliance, moral violation, affective but moral-neutral episode.

	Valence				Arousal				Morality			
	*Mean*	*SD*	*Min*	*Max*	*Mean*	*SD*	*Min*	*Max*	*Mean*	*SD*	*Min*	*Max*
Moral compliance												
Care	7.41	0.43	6.18	8.03	5.69	0.72	4.14	7.14	7.63	0.46	6.63	8.22
Fairness	6.98	0.60	5.02	7.61	5.26	0.48	4.17	6.22	7.26	0.51	5.92	8.35
Authority	6.56	0.68	4.99	7.98	4.60	0.46	3.95	6.01	6.78	0.55	5.43	7.62
Ingroup	6.65	0.75	4.66	8.02	4.98	0.53	4.02	6.04	6.67	0.70	5.63	8.11
Purity	6.76	0.57	5.30	7.51	4.73	0.56	3.70	5.66	7.10	0.52	6.04	7.79
Moral violation												
Harm	1.89	0.34	1.19	2.63	5.98	0.54	4.83	7.01	2.05	0.41	1.35	3.01
Unfairness	2.47	0.70	1.40	4.65	5.06	0.58	3.86	6.42	2.61	0.80	1.45	5.04
Despise	2.77	0.70	1.54	3.97	4.74	0.54	3.88	5.78	3.04	0.87	1.51	4.63
Betrayal	2.94	0.99	1.73	5.17	4.76	0.59	3.59	5.81	3.26	1.06	1.82	5.22
Impurity	2.53	0.72	1.52	4.04	5.18	0.67	3.77	6.44	2.57	0.71	1.57	4.24
Affective but non-moral												
Positive	6.88	0.44	5.89	7.67	4.85	0.57	3.62	5.78	5.83	0.30	5.21	6.43
Negative	3.43	0.42	2.64	4.13	4.61	0.51	3.74	6.09	4.99	0.18	4.59	5.41
Neutral	5.84	0.62	4.76	7.28	3.81	0.59	2.82	4.80	5.71	0.46	4.91	7.18

**Table 3:** Mean degree of relevance to moral categories for each moral compliance, moral violation, affective but moral-neutral episode.

	Degree of relevance to care/harm theme (%)				Degree of relevance to fairness/unfairness theme (%)				Degree of relevance to authority/despise theme (%)				Degree of relevance to ingroup/betrayal theme (%)				Degree of relevance to purity/impurity theme (%)			
	*Mean*	*SD*	*Mm*	*Max*	*Mean*	*SD*	*Min*	*Max*	*Mean*	*SD*	*Min*	*Max*	*Mean*	*SD*	*Min*	*Max*	*Mean*	*SD*	*Min*	*Max*
Moral compliance																				
Care	71.77	6.68	58.57	81.72	56.43	7.00	43.66	71.34	38.65	4.72	28.48	47.76	43.07	7.28	30.36	59.11	49.42	4.96	38.67	57.81
Fairness	48.64	10.98	25.80	71.76	69.78	8.94	46.10	83.07	44.95	8.66	25.80	62.27	55.13	12.14	34.87	79.55	48.46	8.99	34.08	65.42
Authority	38.83	10.14	27.52	62.35	44.70	6.28	32.74	62.41	61.15	7.58	46.17	77.59	57.87	9.04	40.35	74.46	39.00	5.19	26.11	47.65
Ingroup	42.82	11.97	24.67	72.73	53.13	13.47	32.29	83.36	47.58	7.49	33.33	59.43	62.47	9.32	44.86	81.24	43.00	6.83	32.29	62.12
Purity	45.96	11.92	24.96	68.35	43.76	8.86	29.82	61.10	45.74	8.16	32.00	64.09	42.62	7.67	30.96	65.00	65.07	8.05	49.52	79.66
Moral violation																				
Harm	76.85	7.53	56.04	89.90	59.86	7.28	39.04	74.73	47.92	6.15	36.43	65.80	51.10	9.10	32.17	71.57	57.93	7.98	42.50	70.92
Unfairness	52.35	13.11	34.18	80.20	68.20	8.92	48.24	80.31	48.03	9.34	36.11	68.47	59.38	11.25	37.16	80.20	51.79	8.73	35.09	67.14
Despise	46.37	13.59	26.70	67.14	50.31	10.09	33.01	70.38	62.42	7.37	47.26	76.73	57.53	7.67	44.78	77.24	43.96	7.52	30.35	59.04
Betrayal	43.16	11.46	21.48	63.18	54.39	15.52	25.10	79.21	49.56	11.35	30.99	68.30	63.54	13.99	39.49	81.88	45.61	11.08	24.09	66.29
Impurity	53.88	15.46	23.02	86.73	50.64	8.03	35.90	66.33	51.22	9.67	33.49	75.24	49.57	11.54	29.02	75.00	65.70	8.42	47.23	83.06
Affective but non-moral																				
Positive	27.83	7.14	15.00	48.38	30.43	8.35	19.55	62.22	27.11	8.10	13.18	49.73	29.29	10.13	14.20	56.52	30.54	8.57	16.48	51.57
Negative	35.43	9.68	18.48	58.86	25.35	4.96	16.33	39.90	21.76	3.73	13.88	31.37	22.31	4.75	14.69	39.57	24.57	3.73	18.67	31.70
Neutral	24.95	5.17	15.57	42.65	28.28	6.26	16.82	42.96	24.80	6.60	13.64	45.40	27.74	11.36	13.86	63.70	25.99	5.90	15.00	42.50

#### Correlations between valence, arousal, and morality

**Figure 1:**
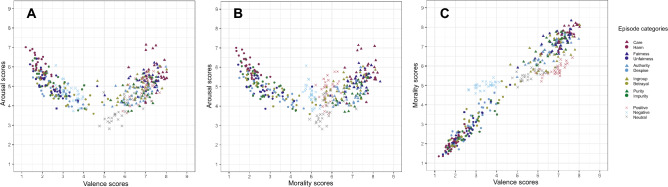
Relationships between mean valence, arousal, and morality scores. (**A**) Relationship between valence and arousal scores. (**B**) Relationship between morality and arousal scores. (**C**) Relationship between valence and morality scores. Each color means the affective categories for episodes.

Figure 1 shows the relationships among valence, arousal, and morality ratings. The relationships between the valence and the arousal ratings (Fig. 1A) and between the morality and the arousal ratings (Fig. 1B) appeared to be boomerang or U-shaped. That is, the arousal ratings were higher when the valence and morality ratings of episodes were at their extreme values. Such boomerang shapes have been generally seen in the relationship between the affective valence and arousal ratings for the images^33–35^. These findings demonstrate that the relationship between morality and arousal ratings is somewhat consistent with the relationship between affective valence and arousal ratings. Figure 1C shows the relationship between the morality and valence ratings. The mean morality ratings seemed to strongly correlate with the mean valence ratings, which mainly contained the moral or the moral violation episode. There also existed an almost horizontal distribution, which contained the affective but moral-neutral episodes. Since the moral compliance and moral violation episodes were created to include the acts related to morality and immorality, but not the affective but moral-neutral episodes, the range of morality ratings for moral compliance and violation episodes may have been wider, resulting in the different shapes of the distributions of included moral compliance and violation episodes from that of included the affective but moral-neutral episodes.

To investigate the associations of ratings of valence, arousal, morality, and relevance of moral categories to the episodes, we performed Pearson correlation analyses (we used the R for analysis; R code is included in the file placed in the OSF). Table 4 shows the correlation coefficients among the ratings of valence, arousal, morality, and relevance of moral categories to the episode. The ratings of morality were highly positively correlated with the ratings of valence, *r* = 0.96, *t*(387) = 63.86, *p* < 0.001 (see also Table 4). There were the negative correlations between arousal and valence ratings, *r* = − 0.13, *t*(387) = − 2.68, *p* = 0.008, and between arousal and morality ratings, *r* = − 0.16, *t*(387) = − 3.27, *p* = 0.001. Moreover, the positive correlations were found between the relevance ratings of all the moral categories. In particular, it was shown that when the relevance of *Fairness* to the episodes was assessed to be higher, the relevance of *Care*, *Ingroup*, and *Purity* to the episodes also tended to be assessed to be higher (*Care*:* r* = 0.64, *t*(387) = 16.35, *p* < 0.001; *Ingroup*: *r* = 0.73, *t*(387) = 21.30, *p* < 0.001; *Purity*:* r* = 0.62, *t*(387) = 15.63, *p* < 0.001). It was also shown that *Purity* category was highly related to *Care* category, *r* = 0.64, *t*(387) = 16.23, *p* < 0.001. Furthermore, *Ingroup* category was highly correlated with *Authority* category,* r* = 0.82, *t*(387) = 28.55, *p* < 0.001. These results indicate that the moral categories of the episodes are not completely independent.

**Table 4:** Correlations between valence, arousal, morality, and ratio of each moral categories for each episode.

	Mean	SD	Arousal	Morality	Degree of relevance to care/harm theme (%)	Degree of relevance to fairness/unfairness theme (%)	Degree of relevance to authority/despise ratio theme (%)	Degree of relevance to ingroup/betrayal ratio theme (%)	Degree of relevance to purity/impurity ratio theme (%)
Valence	4.838	2.17	− 0.13**	0.96***	− 0.26***	− 0.19***	− 0.24***	− 0.2***	− 0.22***
Arousal	4.943	0.76		− 0.16**	0.79***	0.6***	0.33***	0.36***	0.56***
Morality	5.025	2.07			− 0.25***	− 0.2***	− 0.25***	− 0.22***	− 0.24***
Degree of relevance to care/harm theme (%)	46.89	17.84				0.64***	0.38***	0.37***	0.64***
Degree of relevance to fairness/unfairness theme (%)	48.91	16.34					0.58***	0.73***	0.62***
Degree of relevance to authority/despise theme (%)	43.97	14.47						0.82***	0.58***
Degree of relevance to ingroup/betrayal theme (%)	47.87	16.52							0.49***
Degree of relevance to purity/impurity theme (%)	45.51	14.78							

The *p* values were not adjusted in the analysis.

#### Cluster analyses of moral compliance and moral violation episodes by using MAES

By using MAES, we analyzed how many categories the moral compliance and violation episodes could be classified based on their relevance ratings of each category for the episodes.

For determining the best number of clusters, a cluster analysis using NbClust packages in R (method: ward.D2;^36,37^) was conducted for the moral compliance and violation episodes, separately. NbClust is a package that provides the best clustering scheme according to 30 indicators for identifying the number of clusters^36^. This method was selected to ensure that the choice of the number of clusters was not arbitrary. The moral violation episodes were classified into two clusters and the moral compliance episodes were classified into three clusters. Thus, the hierarchical clustering (ward.D2 method) with hclust and partitioning the data by the cutree function according to the optimal number of clusters calculated by the NbClust packages (ward.D2 method) (Table 5).

**Table 5:** Numbers of clusters for each gender or age.

Experiment 1			
Category	Number of clusters	Gender	Number of clusters
Immoral	2	Female	2
		Male	2
Moral	3	Female	6
		Male	2

Category	Number of clusters	Age	Number of clusters
Immoral	2	Emerging adult	2
		Young adult	3
		Middle adult	2
		Late adult	2
Moral	3	Emerging adult	2
		Young adult	4
		Middle adult	4
		Late adult	2

**Figure 2:**
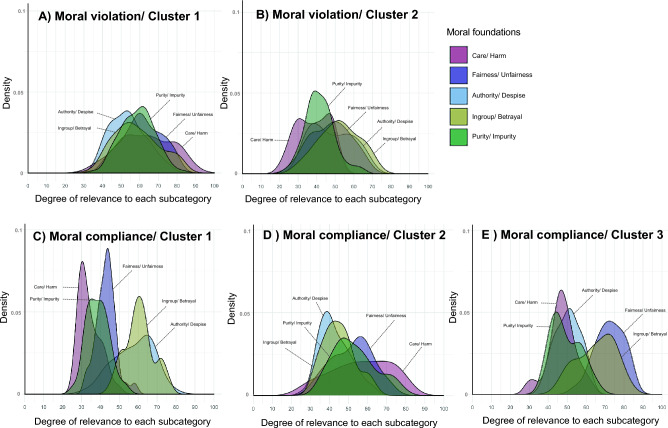
Density of the contents of the five moral categories for each cluster. (**A**) and (**B**) Mean two clusters of the moral violation episodes. (**C**)–(**E**) Mean four clusters of the moral compliance episodes. Each color means five moral categories.

Figure 2 indicates the distributions of the degree of relevance to each moral categories for each cluster of moral compliance or violation episodes. One of the two clusters (i.e., Moral violation/Cluster 1) contained moral violation episodes with low degrees in all moral categories, while the other cluster (i.e., Moral violation/Cluster 2) contained moral violation episodes with high degrees in all moral categories. Our participants might tend to rate equally the relevance of all moral categories for the moral violation episodes. This might indicate that the moral categories for the immoral acts are not exclusive.

For the clusters of moral compliance episodes, the first group (i.e., Moral compliance/Cluster 1) included the episodes with a high degree of *Ingroup* and *Authority* categories, the second group (i.e., Moral compliance/Cluster 2) included the episodes with a high percentage of *Care* category, the third group (i.e., Moral compliance/Cluster 3) included the episodes with a high percentage of *Ingroup* and *Fairness* categories. These suggested that the *Ingroup*, *Authority*, and *Fairness* categories have similar evaluation trends, whereas *Care* category is relatively evaluated independently.

To explore the gender differences in the number of clusters, we divided the data based on reported gender and conducted the cluster analysis for the relevance ratings of each moral category. The results showed that the number of clusters in the moral violation episodes seemed to be relatively stable across genders. On the other hand, the estimated number of clusters in the moral compliance episodes was greater for the female participants than the male participants.

Previous studies suggested that the moral foundations might not be stable across the age of cohorts (e.g.,^38,39^). To examine the effect of ages on the number of clusters, the participants were divided into four ages of cohorts (i.e., Emerging adults, Young adults, Middle adults, and Late adults) in accordance with Sağel^39^. The results of cluster analysis for each age of the cohort showed that the number of clusters was not stable across ages in both moral violation and compliance episodes.

#### Gender differences

**Figure 3:**
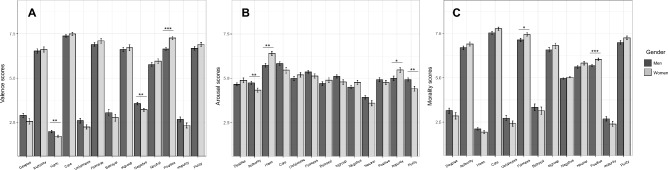
Gender differences of mean valence, arousal and morality scores for each episode’s category. (**A**) Mean valence scores. (**B**) Mean arousal scores. (**C**) Mean morality scores. (***p < .001, **p < .01, *p < .05).

Studies have shown that there may exist gender differences in moral judgments^40,41^. To test whether there were the gender differences of the valence, the arousal, the morality ratings for each category of our episodes, we conducted the Welch’s two-sample *t* test for each these ratings. The *p* values were not adjusted in the analyses as the episodes were qualitatively different. Figure 3 showed the differences of participant gender on valence, arousal, and morality ratings for each category of the episodes. The results showed that our female participants rated negatively more than our male participants in the episodes associated with *Harm* (*female: Mean* = 1.73, *SD* = 0.37*; male: Mean* = 1.99, *SD* = 0.36; *t*(57.95) = 2.85, *p* = 0.006, *d* = − 0.71) and negative contents (*female: Mean* = 3.22, *SD* = 0.46*; male: Mean* = 3.57, *SD* = 0.42; *t*(57.43) = 3.00, *p* = 0.004, *d* = − 0.79). In addition, the female participants rated positively than the male participants for the positive episodes (*female: Mean* = 7.26, *SD* = 0.46*; male: Mean* = 6.64, *SD* = 0.47; *t*(55.92) = 5.08, *p* < 0.001, *d* = 1.33). There were no gender differences in other episode categories (*t* < 1.96, *p*s > 0.05). Regarding the arousal ratings, the results showed that the male participants tended to perceive higher arousal than the female participants for the episodes associated with *Authority* (*female: Mean* = 4.33, *SD* = 0.62*; male: Mean* = 4.74, *SD* = 0.46; *t*(53.49) = 2.93, *p* = 0.005, *d* = − 0.75) and with *Purity* (*female: Mean* = 4.41, *SD* = 0.71*; male: Mean* = 4.93, *SD* = 0.50; *t*(52.09) = 3.26, *p* = 0.002,* d* = − 0.85), and the female participants tended to perceive higher arousal for episodes associated with *Harm* (*female: Mean* = 6.40, *SD* = 0.68*; male: Mean* = 5.73, *SD* = 0.54; *t*(54.98) = − 4.21, *p* < 0.001, *d* = 1.09) and *Impurity* (*female: Mean* = 5.46, *SD* = 0.75*; male: Mean* = 4.99, *SD* = 0.68; *t*(57.47) = 2.53, *p* = 0.014, *d* = 0.66). The differences of gender in other episode categories were not significant (*t* < 1.92, *p*s > 0.06). For the morality ratings, the female participants evaluated as more morally than the male participants for *Fairness* (*women: Mean* = 7.46, *SD* = 0.56*; men: Mean* = 7.15, *SD* = 0.50; *t*(55.24) = 2.24, *p* = 0.029, *d* = 0.58) and the positive episodes (*women: Mean* = 6.05, *SD* = 0.40*; men: Mean* = 5.69, *SD* = 0.31; *t*(52.58) = 3.81, *p* < 0.001, *d* = 1.01). We found no significant difference of gender on the other episode categories (*t* < 1.85, *p*s > 0.07). Thus, while the modality judgments are generally consistent between men and women, there are slight differences in ratings between men and women on some moral foundations.

### Ethics approval

Ethical approval for our study was obtained by the Ethics Review Committee on Research with Human Subjects in Waseda University (No. 2019-357(1)).

### Consent to participate

Informed consent was obtained from all participants included in the study.

### Consent for publication

All participants have consented to the submission of the data to the journal.

### Discussion

We explored the categories of moral violation and moral compliance by developing the Moral and affective episodes set (MAES) and evaluating them. Experiment 1 showed that the moral compliance episodes were divided into three clusters, whereas the moral violation episodes were divided into two clusters, suggesting that the frameworks in judging moral compliance and moral violation might be different.

Moral violation episodes seemed to be grouped by the perceived levels of immorality for the episodes, rather than the moral categories. This suggests that moral violation behaviors are being judged based on a single framework (i.e., degree of immorality) rather than based on multiple moral foundations. On the other hand, the moral compliance episodes seemed to be clustered by more than two categories. It is considered that the plurality of moral judgments may fit the structure of moral compliance rather than that the structure of moral violation.

For the episodes of moral compliance, we observed three clusters as follows (i.e., Cluster 1: highly related to *Ingroup* and *Authority*; Cluster 2: highly related to *Care*; Cluster 3: highly related to *Ingroup* and *Fairness*). The relevance ratings especially for *Care* seemed to change independently of other moral bases. Such independence of *Care* is also in line with previous studies and intuitions. For example, several studies arguing for the universality of moral rules have considered the harm component to be particularly crucial in moral judgments and have focused on harm as a basic concept (e.g.,^14,15,42^). Our results also showed that the moral episodes with higher relevance ratings of *Ingroup* also had higher relevance ratings of *Authority* or *Fairness*, suggesting that the judgments about the moral events related to *Ingroup* could affect the judgments about *Authority* or *Fairness*. Consistent with our findings, the previous studies supposed that the categories of ingroup/loyalty and authority/respect could be grouped into one category as the ethic related to the community and groups^19,23^. In addition, the relevance ratings of *Fairness* change with the relevance ratings of *Ingroup* but not the relevance ratings of *Authority*; thus, it is possible that the moral categories of *Ingroup* and *Authority* is partially, rather than completely, overlap.

*Fairness* and *Ingroup* have been assigned into different ethics types in previous taxonomies of morality (e.g., ethics of autonomy and community^13,23^; rights of individuals and values of group unity^19^); however, our findings showed that the relevance ratings about *Fairness* changed along with the relevance ratings of *Ingroup*. One possible explanation is that the episodes about *Fairness* sometimes included words associated with the groups (i.e., company or family); thus, the episodes about *Fairness* might be evaluated as having a higher association with the loyalty for the groups. Also, all participants in the present study were recruited through the Japanese crowdsourcing website and therefore most of them were Japanese. It has been reported that Asian cultures were placed relatively high importance on ethics associated with collectivism^43^. When the peoples with high collectivist tendencies evaluate the episodes about *Fairness*, it may be that they focus on the collectivist issues, rather than the individualistic issues in episodes.

Our results indicated that there were different sensitivities to the moral categories depending on gender. Specially, the female participants rated the events related to *Harm* as more negative than the male participants. On the other hand, the male participants evaluated the events related to *Authority* as leading to more arousal than the female participants. These findings are in line with to the previous studies that suggested that women judged severely for the acts related to *Care*, *Fairness*, and *Purity*, whereas men judged severely for the acts related to *Authority* and *Ingroup* (e.g.,^40,41^). To date, the differences in gender identity can be explained by the intertwined effects of nature and nurture^44^. For the effects of nurture, Niazi, Inam, and Akhtar^45^ examined the effects of gender stereotypes on the predictions of moral ratings and suggested that there is a stereotype that women estimate that men are less sensitive to the moral category of *Care*. Furthermore, since the participants were asked to enter their gender before the survey began in the present study, these procedures may have facilitated attention to their own gender, which might influence their moral judgments for the events. Further research will be needed to determine how moral judgments change depending on what identity of their own they direct their attention.

## Experiment 2

In Experiment 2, the survey was conducted to confirm the reproducibility of the results in Experiment 1. We recruited 1600 participants through the Yahoo crowdsourcing system (https://crowdsourcing.yahoo.co.jp/; April 18th to May 6th, 2024). The data were collected to match the gender and age distribution as closely as possible. If the participants answered incorrectly on a basic quiz about the categories of morality, we judged that the participants did not fully understand the survey and the data were excluded from further analysis. Finally, 1265 participants’ data (648 women, 599 men, others 4, unknown 3, no answer 11, mean age = 43.78 years, age range = 18–80 years) were used in Experiment 2.

The procedure is identical to Experiment 1 except one episode’s contents. Because the parts of technical problems in Experiment 1 was corrected, the number of *Positive* episodes was changed from 29 to 30.

### Results

**Figure 4:**
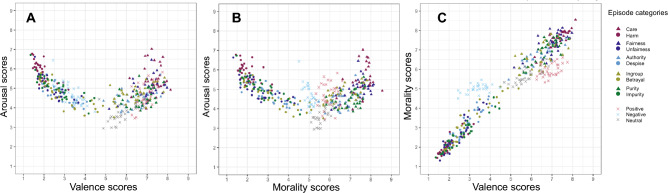
Relationships between mean valence, arousal, and morality scores. (**A**) Relationship between valence and arousal scores. (**B**) Relationship between morality and arousal scores. (**C**) Relationship between valence and morality scores. Each color means the affective categories for episodes.

Table 6 shows the demographics of Experiment 2. Tables 7 and 8 summarize the descriptive statistics for the episodes in Experiment 2. For the relationships between the arousal ratings and affective valence or morality, the results also showed the boomerang shapes (see Fig. 4).

**Table 6:** Basic demographics in Experiment 2.

Experiment 2	*n*	Range	*Mean*	*Median*	*SD*	Gender				
						Women	Men	Others	Unknown	No answer
Emerging adult	269	18–25	21.94	22	2.19	165	93	3	2	6
Young adult	335	26–39	34.67	36	3.53	159	172	1	1	2
Middle adult	352	40–59	50.1	51	5.65	177	172	0	0	3
Late adult	309	60–80	65.45	64`	4.84	147	162	0	0	0
Total	1265		43.78	42	16.38	648	599	4	3	11

**Table 7:** Mean valence, arousal and morality for each moral compliance, moral violation, affective but moral-neutral episode.

	Valence				Arousal				Morality			
	*Mean*	*SD*	*Min*	*Max*	*Mean*	*SD*	*Min*	*Max*	*Mean*	*SD*	*Min*	*Max*
Moral compliance												
Care	7.32	0.45	6.15	8.14	5.45	0.77	3.96	7.03	7.62	0.44	6.34	8.56
Fairness	7.01	0.62	5.16	7.97	5.02	0.56	3.94	6.47	7.32	0.58	6.09	8.14
Authority	6.45	0.73	4.89	7.83	4.45	0.43	3.76	5.72	6.73	0.54	5.47	7.63
Ingroup	6.49	0.73	5.21	7.85	4.71	0.47	3.51	5.55	6.66	0.65	5.70	7.82
Purity	6.75	0.58	5.62	7.81	4.53	0.45	3.86	5.64	7.01	0.55	5.83	7.73
Moral violation												
Harm	1.91	0.43	1.41	3.19	5.86	0.54	4.51	6.79	2.04	0.46	1.44	3.20
Unfairness	2.41	0.60	1.56	3.82	5.04	0.56	4.27	6.63	2.56	0.78	1.31	4.29
Despise	2.84	0.67	1.90	4.08	4.73	0.49	3.95	5.73	3.11	0.86	1.68	4.61
Betrayal	3.00	1.04	1.78	5.37	4.69	0.63	3.60	5.79	3.23	1.11	1.82	5.10
Impurity	2.61	0.82	1.34	4.44	5.08	0.58	3.71	6.73	2.67	0.77	1.47	4.56
Affective but non-moral												
Positive	6.95	0.39	6.25	7.75	4.69	0.64	3.47	5.84	5.94	0.34	5.34	6.61
Negative	3.39	0.48	2.44	4.47	4.69	0.55	3.80	6.44	5.01	0.21	4.51	5.46
Neutral	5.85	0.59	4.67	6.86	3.74	0.47	2.95	4.66	5.75	0.48	5.10	7.37

**Table 8:** Mean degree of relevance to moral categories for each moral compliance, moral violation, affective but moral-neutral episode.

	Care/harm ratio (%)				Fairness/unfairness ratio (%)				Authority/despise ratio (%)				Ingroup/betrayal ratio (%)				Purity/impurity ratio (%)			
	*Mean*	*SD*	*Min*	*Max*	*Mean*	*SD*	*Min*	*Max*	*Mean*	*SD*	*Min*	*Max*	*Mean*	*SD*	*Min*	*Max*	*Mean*	*SD*	*Min*	*Max*
Moral compliance																				
Care	72.55	6.11	60.00	84.43	56.04	7.71	42.07	73.12	37.65	5.14	27.12	48.53	41.27	7.37	27.63	57.88	47.80	6.40	32.72	61.14
Fairness	46.36	11.96	22.37	66.86	70.17	9.05	47.70	83.81	43.76	9.11	25.59	64.29	54.67	12.76	33.90	76.04	45.58	7.05	33.05	61.55
Authority	37.22	10.69	26.19	65.44	42.80	5.89	35.68	65.98	60.94	9.17	37.63	76.19	57.84	9.70	38.47	72.37	36.84	5.65	28.87	53.14
Ingroup	41.53	11.99	21.63	68.98	52.25	13.66	25.87	83.37	44.81	7.08	30.00	62.29	61.23	8.84	47.17	76.61	40.33	6.42	31.24	58.70
Purity	42.87	10.86	25.87	64.84	41.57	8.90	28.26	57.74	42.88	6.84	31.36	59.01	39.74	8.50	27.74	63.64	62.35	8.89	48.08	80.00
Moral violation																				
Harm	75.57	7.09	61.13	85.71	55.86	7.63	39.29	67.58	45.95	6.81	35.82	61.52	48.51	10.04	30.00	70.67	54.44	7.70	39.38	70.14
Unfairness	49.94	12.81	29.84	79.43	66.56	10.16	41.77	81.43	47.06	8.68	33.89	67.43	58.12	11.19	36.29	79.43	48.87	9.07	30.72	67.14
Despise	45.15	14.35	26.37	79.03	49.74	10.38	35.23	72.32	61.00	7.46	42.57	74.03	55.92	8.09	44.30	72.61	42.79	8.19	30.22	54.95
Betrayal	41.32	12.58	20.34	63.70	52.47	15.61	23.57	76.42	47.70	11.82	26.92	61.65	62.23	14.70	34.86	86.39	42.52	10.75	22.88	58.15
Impurity	51.86	16.20	21.03	84.43	47.65	10.45	27.20	68.57	49.35	9.99	34.60	72.74	48.36	12.92	27.11	75.00	62.78	9.78	41.27	81.57
Affective but non-moral																				
Positive	26.52	6.69	14.58	41.50	29.62	7.72	16.10	59.07	26.79	8.47	14.24	55.33	27.96	9.46	15.25	51.44	29.76	7.43	17.97	55.24
Negative	35.01	11.72	19.72	65.89	24.56	5.18	17.43	38.21	21.71	3.80	14.71	32.00	22.19	5.65	15.14	40.28	24.71	4.33	19.09	36.81
Neutral	24.67	4.93	15.57	41.86	28.14	6.17	19.15	43.51	25.07	6.95	17.29	47.97	27.01	10.96	16.95	62.43	26.39	6.30	18.66	46.19

#### Correlations between valence, arousal, and morality

We performed Pearson correlation analyses to examine the relationships among the ratings of valence, arousal, morality, and relevance of moral categories to the episode. The results showed in Table 9. The overall trend of results was identical to that of Experiment 1.

**Table 9:** Correlations between valence, arousal, morality, and ratio of each moral category for each episode.

	*Mean*	*SD*	Arousal	Morality	Degree of relevance to Care/harm theme (%)	Degree of relevance to Fairness/unfairness theme (%)	Degree of relevance to Authority/despise ratio theme (%)	Degree of relevance to Ingroup/betrayal ratio theme (%)	Degree of relevance to Purity/impurity ratio theme (%)
Valence	4.833	2.14	− 0.25***	0.95***	− 0.26***	− 0.16***	− 0.24***	− 0.21***	− 0.22***
Arousal	4.823	0.73		− 0.26***	0.77***	0.55***	0.31***	0.34***	0.52***
Morality	5.038	2.07			− 0.25***	− 0.16***	− 0.26***	− 0.23***	− 0.25***
Degree of relevance to Care/harm theme (%)	45.44	18.13				0.59***	0.35***	0.33***	0.60***
Degree of relevance to Fairness/unfairness theme (%)	47.49	16.42					0.55***	0.72***	0.56***
Degree of relevance to Authority/despise theme (%)	42.67	14.20						0.81***	0.54***
Degree of relevance to Ingroup/betrayal theme (%)	46.55	16.69							0.44***
Degree of relevance to Purity/impurity theme (%)	43.48	14.00							

#### Cluster analyses of moral compliance and moral violation episodes

Utilizing the episode data from Experiment 2, we analyzed how many categories the moral compliance and violation episodes could be clustered into. The analysis methods were identical to Experiment 1. The analysis for determining the best number of clusters resulted that the moral violation episodes were classified into three clusters and the moral compliance episodes were classified into four clusters. Therefore, the moral violation and compliance episodes were partitioning by the cutree function according to each optimal number of clusters.

**Figure 5:**
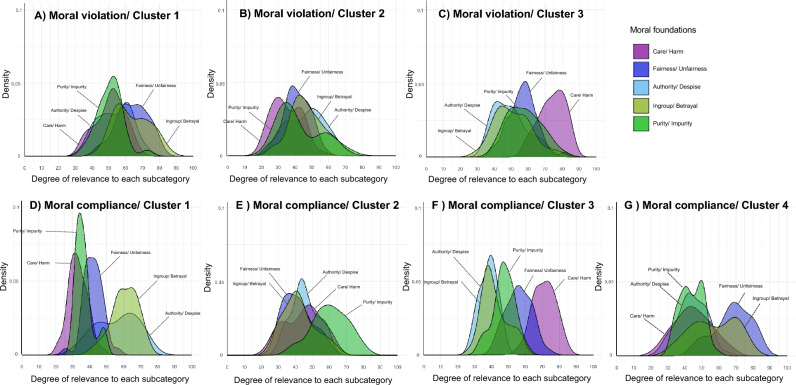
Density of the contents of the five moral categories for each cluster. (**A**)–(**C**) Mean three clusters of the moral violation episodes. (**D**)–(**G**) Mean four clusters of the moral compliance episodes. Each color means five moral categories.

Figure 5 shows the distributions of the relevance to each five moral categories for each cluster of moral compliance or violation episodes. The distributions of all moral categories in moral violation in Cluster 1 were relatively located at the average ratios. The moral violation episodes in Cluster 2 contained episodes with low degrees of relevance to all moral categories, compared to Cluster 1. Further, the moral violation episodes in Cluster 3 included the episodes with a high degree of *Harm* category. These results were partially overlapped with the results in Experiment 1. It seems that there is a bias to respond equally to the relevance of all moral categories for the moral violation episodes. However, as seen in Cluster 3, there were judgments in which especially *Harm* was rated strongly.

The number of clusters in moral compliance episodes was four; Cluster 1 of moral compliance episodes contained the episodes with a high degree of *Ingroup* and *Authority* categories, Cluster 2 of moral compliance episodes had the episodes with a high percentage of *Purity* category, Cluster 3 of moral compliance episodes included the episodes with a high percentage of *Care* category, Cluster 4 of moral compliance episodes included the episodes with a high percentage of *Fairness* and *Ingroup* categories. These results showed that the judgments about *Ingroup*, *Authority*, and *Fairness* are similar, whereas the *Care* or *Purity* category is judged independently for the specific episodes.

Table 10 shows the results of cluster analysis by each gender (only responses from women or men were analyzed for the cluster analysis) and age. Results showed that there were no differences in the cluster numbers across the genders in both moral violation and compliance episodes. For the age-related differences, the number of clusters was two or three across age cohorts in moral violation episodes. For the moral compliance episodes, the number of clusters was not stable across age cohorts.

**Table 10:** Number of clusters for each gender or age.

Experiment 2			
Category	Number of clusters	Gender	Number of clusters
Immoral	3	Female	3
		Male	3
Moral	4	Female	4
		Male	4

Category	Number of clusters	Age	Number of clusters
Immoral	3	Emerging adult	3
		Young adult	2
		Middle adult	2
		Late adult	2
Moral	4	Emerging adult	2
		Young adult	6
		Middle adult	3
		Late adult	2

### Discussion

We conducted Experiment 2 to confirm the reproducibility of the results in Experiment 1. Results indicate that the moral compliance episodes have four clusters, whereas the moral violation episodes provide three clusters. The number of clusters in Experiment 2 was different from those in Experiment 1. Specifically, the moral violation episodes that are independently judged for *Harm* and the moral compliance episodes that are rated independently for *Purity* were found in Experiment 2.

## General discussion

We examined the extent to which moral judgments are exclusive across moral categories and explored whether the framework of judgments for moral violation and compliance would be different. The present study demonstrated that some moral judgments such as care/harm are relatively exclusive across moral categories. Also, regarding moral compliance behaviors, authority-related or fairness-related judgments appeared to be easily mixed with ingroup-related judgments. Furthermore, our findings showed that the number of clusters was different between moral violation and moral compliance episodes. Specifically, the number of clusters in moral compliance episodes was greater than those in moral violation episodes. It is possible that the moral judgments for the moral violation episodes were borderless across moral categories compared to those for the moral compliance episodes (Supplementary Material).

Both experiments suggested that moral violations are somewhat judged based on a single framework (i.e., degree of immorality) compared to moral compliance. One possible explanation concerns the generalization of negative responses. In the context of fear learning, it was known that various objects that resemble fear conditioned objects also elicit fear (i.e., fear generalization^46^). Similarity, it is possible that negative responses for specific moral violated acts spread to responses for other moral violated acts. Further studies should examine how the impact of negative emotions for the moral violation acts would confuse classification of mora acts.

We found that the number of clusters was not fixed across experiments, suggesting that the structures of moral judgments might not be unchanged. The data from Experiment 1 was collected during the period of impact of COVID-19, whereas the data of Experiment 2 was gathered after the declaration of a state of emergency about COVID-19. Aftereffects of the threat for infections might affect moral judgments. In accordance with the findings, the several studies pointed out the possibility that the moral bases were not be stable across time and susceptible to flexible cognitive and emotional states^38,47^.

There exist some limitations in this study that need to be considered (Table 11). First, the participants were the people in Japan; therefore, our findings may be specific to the present samples. Future studies should examine whether the differences between moral violation and moral compliance will be generalized in other countries. If there are differences in the categorization for moral violation and/or moral compliance among countries, the moral-related beliefs may be modulated by the rules and customs in the countries or environments where people have interacted. Furthermore, previous studies pointed out that the political ideology (i.e., liberals and conservatives) would affect the moral judgments and the configurations of the five moral foundations (e.g.,^19^). Brown, Chua, and Lukaszewski^48^ suggested that the socioeconomic status was related to the endorsing “binding” foundations (loyalty, purity, respect for authority). Further studies should focus on how the political and socioeconomic status influence the clusters of moral judgments. The second concern is that we used the cluster analysis for the relevance ratings for moral categories. Other analyses should be utilized to test for generalizability; for example, further insights might be gained by natural language processing for the episodes set. Third, using episodes contained the contents of rules and place names in Japan and/or Asia. Whether the observed moral categories in this study are found in other countries and environments may depend on the understanding of the episodes. Further studies should explore whether the findings depend on the understanding of the episodes. Moreover, if one tries to verify these episodes in other countries, it may be difficult to understand the meanings of the episodes. For further studies, we placed the English versions of episodes set in OSF that were marked to the episodes that included Japanese and/or Asian-specific names and rules. Several previous studies suggested that the moral judgments are affected by wordings (e.g., “always”) and framings^49–52^. The present study did not confirm whether the episodes had clear thematic and linguistic distinctions between moral categories. Also, it should be noted that the possibility that the episodes were unintentionally biased by the author cannot be rejected. A future study should examine whether the structures of moral violation and compliance would be different even when the wording are controlled. The last point concerns the type of stimuli. The richness of information may differ depending on the type of stimulus, such as words, episodes, images, and videos. Future studies should address whether the differences of judgments in moral violation and moral compliance differ among the types of stimuli.

**Table 11:** Limitation table in this study.

Limitations	Reproducibility and generalizability, different settings
Samples	People in Japan participated in our study. Future study should examine whether this finding is also observed in other countries. We translated the episodes to English and placed the English versions of episodes set in OSF
Analysis	Cluster analysis were conducted for the ratings for five moral categories. Future study should also explore using other types of analysis (e.g., language processing for the episodes)
Stimuli type	We used the episodes; but the moral category may be different depending on the types of stimuli (e.g., movie, images)
Stimuli contents	Some episodes may include the names and social rules in Japan/Asia. We prepare the files that marked the episodes that may contain the Japanese/Asian place names or rules

In summary, we developed the moral and affective episodes set (MAES) and found that the frameworks of judgments of morality and immorality would be different. Our findings point to the importance of considering not only on moral violation, but also on moral compliance for further understanding the structure of the moral foundations. The MAES provides the ratings of affective valence, arousal, morality, and relevance of each moral foundation for 389 moral compliance and violation episodes. It would be interesting and important to test the validity of the MAES in different populations. The MAES with all the ratings can be freely available for research purpose through this URL (OSF, https://osf.io/jtxyp/?view_only=63607826eed24593a4a7c7f0337b0b62).

### Supplementary Information

Supplementary Information.

## Data Availability

The datasets generated during and/or analyzed during the current study are available in the OSF repository, [https://osf.io/jtxyp/?view_only=63607826eed24593a4a7c7f0337b0b62].
