# Supplementary material for: Different judgment frameworks for moral compliance and moral violation
Source: Sci Rep. 2024 Jul 16;14:16432. doi: 10.1038/s41598-024-66862-9 (PMC11252160; doi:10.1038/s41598-024-66862-9)
Supplement: Supplementary file 1 — Supplementary Information. [file 41598_2024_66862_MOESM1_ESM.docx]

**Supplementary Material: Question items displayed in the research.**

**[Question 1]** How do you feel the negative or positive emotions when you read the episode? Please choose the number from 1 (i.e., “I feel very negative emotions”) to 9 (i.e., “I feel very positive emotions”) that most closely matches your feelings. ※ Please answer not whether “this person” in the episode felt negative or positive, but whether you yourself felt negative or positive after reading this episode. Also, please evaluate the episode based on how you felt about it, not on how it would be evaluated in general.

**[Question 2]** How do you feel the arousal after reading the episode? Please choose the number that most closely matches your feelings from 1 to 9, ranging from “1. Very low arousal” to “9. Very high arousal”.

※ “Arousal” indicates the degree to which you feel excitement, such as surprise, agitation, and pounding heart. The higher the number from 1 to 9, the higher the degree of arousal expressed. The degree of arousal has nothing to do with the answer to [Question 1], and the degree of arousal can be higher (closer to the number 9) or lower (closer to the number 1) even if the episode has a positive meaning. Conversely, the degree of arousal may be higher (closer to the number 9) or lower (closer to the number 1) even if the episode has a negative meaning.

**[Question3]** Do you feel that the actions of “this person” in the episode are immoral or moral? Please choose the number that most closely matches your feelings from 1 to 9, ranging from “1. Extremely immoral” to “9. Extremely moral”. ※ Please evaluate this question based on how you feel about it, not on how it is likely to be evaluated in general.

**[Question 4]** How much do you feel this episode is related to each of the following five themes? Please answer from 0 to 100% for each. Please note that you do not have to add all of the following together to make 100%. Please indicate how many percentages you feel the following themes are related to the episode, individually.

**[Question 5] *Themes related to* “*Care/Harm*”**

“*Care/Harm*” is related to protecting and caring for the weak without harming or causing pain. If you feel that the episode contains themes related to “*Care/Harm*”, please describe the extent of it using percentages. In this case, both protecting and harming others are episodes related to “*Care/Harm*”, so the percentages will be larger.

**[Question 6] *Themes related to* “*Fairness/*** ***Reciprocity*”**

The theme of “*Fairness/ Reciprocity*” is related to "mutual cooperation and justice without injustice or deception. If you feel the episode contains themes related to “*Fairness/ Reciprocity*”, please describe the extent of these themes using percentages. In this case, the percentages will be larger because the episodes are related to “*Fairness/ Reciprocity*” in both cases of observing and violating fairness.

**[Question 7] *Themes related to* “*Ingroup/ Loyalty*”**

“*Ingroup/* *Loyalty*” is related to being loyal to the group to which one belongs and fulfilling one's duties and contributing to the group without betrayal. If you feel that the episode contains themes related to “*Ingroup/ Loyalty*”, please express the extent of these themes using percentages. In this case, both contributing to the group and betraying the group are episodes related to “*Ingroup/* *Loyalty”*, so the percentage should be large.

**[Question 8] *Themes related to “Authority / Respect”***

“*Authority/ Respect*” is related to respecting hierarchical relationships in order to maintain social order without neglecting hierarchical relationships. If you feel that the episode contains themes related to “*Authority/ Respect*”, please express the extent of these themes using percentages. In this case, both respectful and disrespectful attitudes toward authority (e.g., the president of a company, the patriarch of a family, etc.) will be considered episodes related to “*Authority/ Respect*”, so the percentages will be larger.

**[Question 9] *Themes related to* “*Purity/*** ***Sanctity*”**

The theme of “*Purity/* *Sanctity*” is related to the abstinence from physical and mental defilement, and the preservation of purity and chastity. If you feel that the episode contains themes related to “*Purity/ Sanctity*”, please describe the extent of it using percentages. In this case, both keeping clean and defiling are episodes related to “*Purity/ Sanctity*”, so the percentages are larger.
